# WGS-Based Phenotyping and Molecular Characterization of the Resistome, Virulome and Plasmid Replicons in *Klebsiella pneumoniae* Isolates from Powdered Milk Produced in Germany

**DOI:** 10.3390/microorganisms10030564

**Published:** 2022-03-05

**Authors:** Gamal Wareth, Jörg Linde, Philipp Hammer, Mathias W. Pletz, Heinrich Neubauer, Lisa D. Sprague

**Affiliations:** 1 Friedrich-Loeffler-Institut, Institute of Bacterial Infections and Zoonoses (IBIZ), Naumburger Str. 96a, 07743 Jena, Germany; joerg.linde@fli.de (J.L.); heinrich.neubauer@fli.de (H.N.); lisa.sprague@fli.de (L.D.S.); 2 Institute for Infectious Diseases and Infection Control, Jena University Hospital, Jena University, 07743 Jena, Germany; mathias.pletz@med.uni-jena.de; 3 Department of Bacteriology, Immunology and Mycology, Faculty of Veterinary Medicine, Benha University, Toukh 13736, Egypt; 4 Department of Microbiology and Biotechnology, Max Rubner-Institut, 24103 Kiel, Germany; philipp.hammer@mri.bund.de; 5 Research Campus Infectognostics, Philosophenweg 7, 07743 Jena, Germany

**Keywords:** *Klebsiella pneumoniae*, WGS, resistome, plasmidome, virulome, powdered milk, Germany

## Abstract

The emergence of *Klebsiella pneumoniae* (*K. pneumoniae*) in German healthcare is worrying. It is not well-investigated in the veterinary world and food chains. In the current study, antibiotic susceptibility profiles of 24 *K. pneumoniae* strains isolated from powdered milk samples produced in Germany were investigated by a microdilution test. Next-generation sequencing (NGS) was applied to identify genomic determinants for antimicrobial resistance (AMR), virulence-associated genes and plasmids replicons. All isolates were susceptible to the majority (14/18) of tested antibiotics. Resistance to colistin, fosfomycin, chloramphenicol and piperacillin was found. The ambler class A ß-lactamase, *bla*_SHV_ variants were identified in all isolates, of which *bla*_SHV-187_ was most prevalent and found in 50% of isolates. Single-nucleotide-variants of *oqx*A and *oqx*B conferring resistance to phenicol/quinolone were found in all isolates, and the *oqx*B17 was the most prevalent found in 46% of isolates. 67% of isolates harbored *fos*A genes; however, only one was fosfomycin-resistant. Two isolates harbored genes conferring resistance to colistin, despite being susceptible. The majority of identified virulome genes were iron uptake siderophores. Two enterobactins (*ent*B, *fep*C), six adherence-related genes belonging to *E. coli* common pilus (ECP) and one secretion system (*omp*A gene) were found in all isolates. In contrast, yersiniabactin was found in two isolates. One ST23 strain was susceptible to all tested antibiotics, and harbored determinants discriminatory for hypervirulent strains, e.g., aerobactin, salmochelin, yersiniabactin, enterobactin and regulator of mucoid phenotype A genes that are highly associated with hypervirulent *K. pneumoniae*. The IncF plasmid family was found in all strains, while almost half of the isolates harbored Col440I-type plasmids and nine isolates harbored various Inc-type plasmids. The presence of *K. pneumoniae* carrying different resistomes and major virulent specific virulomes in powdered milk samples is alarming. This could threaten public health, particularly of neonates and infants consuming dried milk.

## 1. Introduction

*Klebsiella pneumoniae* (*K. pneumoniae*) is a Gram-negative, non-motile, usually capsule-forming bacterium. The opportunistic pathogen is associated with severe infections and high mortality rates in intensive care unit (ICU) patients [1]. It is one of the ‘ESKAPE’ pathogens, the most common and serious multidrug-resistant (MDR) pathogen in hospitalized patients worldwide [2]. *K. pneumoniae* has been found to be involved in a wide variety of nosocomial infections, such as sepsis, respiratory and urinary tract infections and wound infections [3]. It has also been isolated from a wide variety of foods, environmental sources such as soil, water and vegetation and dairy farm milk [4]. In recent years, a high prevalence of MDR *K. pneumoniae* (MDR-KP) and carbapenem-resistant *K. pneumoniae* (CRKP) has been observed in hospitalized persons worldwide, and has been associated with unprecedented public health problems [5]. *K. pneumoniae* has been frequently associated with mastitis in bovines [6], leading to high milk production loss, decreased quality and even high mortalities among affected cows [7,8]. The prevalence of this potentially lethal pathogen has been gradually increasing in dairy farms, the environment and the food chain [9].

In Germany, *K. pneumoniae* isolates developed resistance to third-generation cephalosporins and aminoglycosides in the late 20th century [10]. The high incidence of MDR strains has been confirmed in most German federal states [11,12]. The emergence of extended-spectrum beta-lactamases (ESBL) producing *K. pneumoniae* strains is on the rise in Germany, where *bla*_CTX-M-15_, *bla*_CTX-M-3,_ *bla*_SHV-11 and_ *bla*_SHV-5_ are the most frequently detected genes [13,14,15,16]. *K. pneumoniae* has been extensively investigated in humans and hypervirulent (hvKp) and extensively drug-resistant (XDR) strains have been isolated from patients [17,18]. However, research on *K. pneumoniae* is still an exception in veterinary and environmental health, and the risk of human infection regarding food consumption is only just under investigation [19]. Different virulence and pathogenicity factors contribute to *K. pneumoniae* pathogenesis and mediate infectivity, e.g., by means of adherence factors, siderophore activity, O-lipopolysaccharide (LPS) and K-capsular antigens [20]. However, many aspects of its pathogenicity are not yet clear.

The current study aimed at the phenotype and genomic characterization of antimicrobial resistance determinants, pathogenicity-associated genes and plasmid replicons in *K. pneumoniae* strains isolated from milk powder produced in Germany utilizing Next-generation sequence (NGS) technology.

## 2. Materials and Methods

### 2.1. Bacterial Isolates and Identification

All *Klebsiella* spp. isolates used in this study originated from dried milk produced via spray drying (20%) or rotating drum drying (80%) from two companies in Germany. Samples were collected from the end of the production line between 2005 and 2011, as proposed previously [21]. Samples of 10 g were briefly taken by the quality control laboratory of the producers, and tested for the presence of enterobacteria: Bacterial enrichment was carried out in double-buffered peptone water [peptone water 20.0 g L^−l^ (Oxoid, Wesel, Germany), 3.5 g L^−l^ Na_2_HPO_4_ (Merck), 1.5 g L^−l^ KH_2_PO_4_ (Merck)] for 24 h at 30 °C with subsequent plating onto violet red bile dextrose (VRBD) (Merck KGaA, Darmstadt, Germany) agar and incubation for 24 h at 30 °C. VRBD plates showing bacterial growth were transferred to the Max Rubner-Institute. Colonies with different morphotypes were picked and phenotypically characterized by Gram-staining and testing by API 20E (BioMérieux, Nürtingen, Germany). Twenty-four *K. pneumoniae* strains isolated from powdered milk were sent to the Institute of Bacterial Infections and Zoonoses (IBIZ, Jena, Germany) for confirmation and typing. Samples metadata are shown in the Supplementary Table S1. All isolates were re-identified using Matrix-Assisted Laser Desorption/Ionization Time-of-Flight Mass Spectrometry (MALDI-TOF MS) at the species level with a log value > 2.300. Protein extraction from pure colonies of each sample was done as previously described [22]. The MALDI-TOF measurements were carried out using a Microflex LT instrument (Bruker Daltonics, Bremen, Germany), following the MALDI Biotyper manufacturer’s recommendation on the log score value of 0–3 for species identification. Score values between 2.300 and 3.000 were considered ‘highly probable for species identification’; values between 2.000 and 2.290 were considered ‘secure genus identification; values between 1.700 and 1.990 were considered ‘probable genus identification’ and values between 0 and 1.690 were not considered for identification. Whole-genome sequencing data were used to confirm the identity of the genera and the species of each strain using Kraken (v2.0.7_beta) [23]. Kraken 2 (v2.0.7_beta) in combination with the database MiniKraken (v2) were used to classify reads and assemblies and to check for possible contamination. At the genus and species levels, the first match (largest percentage proportion) was considered for identification.

### 2.2. Antibiotics Susceptibility Testing (AST)

The minimum inhibitory concentration (MIC) was determined by the broth microdilution method using an automated MICRONAUT-S system (Micronaut, MERLIN Diagnostics GmbH, Bornheim-Hersel, Germany) and MICRONAUT-S MDR MRGN-Screening MIC plates (Catalog Nr. E1-114-040) as previously described [24]. Strains were automatically classified as susceptible, intermediate and resistant by the MICRONAUTS software, which applies the MIC values according to the Clinical and Laboratory Standards Institute (CLSI, 2020) breakpoint guidelines available for *K. pneumoniae* [25]. The sensitivity of isolates was determined from a panel of 18 antibiotics at different concertations on the plates: ciprofloxacin (CIP), levofloxacin (LEV), amikacin (AMK), chloramphenicol (CMP), fosfomycin (FOS), tigecycline (TGC), colistin (COL), trimethoprim/sulfamethoxazole (T/S), piperacillin (PIP), piperacillin/tazobactam (PIT), cefotaxime (CTX), ceftazidime (CAZ), ceftazidime/avibactam (CAA), ceftolozane/tazobactam (CTA), cefepime (CEP), imipenem (IMP), meropenem (MER) and ertapenem (ERT).

### 2.3. WGS and In-Silico Detection of AMR Determinants, Virulome Genes and Plasmidome

Genomic DNA was extracted using the High Pure PCR Template Preparation Kit (Roche Diagnostics GmbH, Mannheim, Germany) according to the manufacturer’s instructions. Sequencing libraries were prepared with the Nextera XT DNA Library Prep Kit (Illumina, Inc., San Diego, CA, USA), followed by paired-end sequencing on an Illumina MiSeq sequencer (Illumina). To analyze raw sequencing data, the Linux-based pipeline WGSBAC (v2.0, https://gitlab.com/FLI_Bioinfo/WGSBAC, accessed on 28 February 2022) was used as previously described [24,26]. The pipeline performs a quality control of raw sequencing data utilizing FastQC (v0.11.7) files [27], and calculates raw sequencing coverage. For assembly, the pipeline uses Shovill (v1.0.4, https://github.com/tseemann/shovill, accessed on 28 February 2022), which is based on the SPAdes assembler [28]. The quality of the assembled genomes is checked with QUAST (v. 45.0.2) [29]. To identify contaminations, WGSBAC uses the sequence classifier Kraken 2 v1.1 [23] and the database Kraken2DB. For the prediction of virulence factors, WGSBAC utilizes ABRIcate (v0.8.10, https://github.com/tseemann/abricate, accessed on 28 February 2022) together with the Virulence Factor Database [30]. For the determination of genetic features leading to AMR, ABRIcate, together with the databases, Comprehensive Antibiotic Resistance Database (CARD) [31] and ResFinder [32], were applied. In addition, NCBI’s AMRFinderPlus tool [33] was used, together with the *Klebsiella*-specific parameters, to identify point mutations leading to AMR. Abricate against the PlasmidFinder database [34] was used to identify the presence of plasmid replicons. In-silico determination of classical multilocus sequence typing (MLST) was performed by WGSBAC based on the assembled genomes using the software mlst (v2.16.1, https://github.com/tseemann/mlst, accessed on 28 February 2022), which incorporates the species-specific scheme (*K. pneumoniae*). To construct a phylogenetic tree, Snippy (v4.3.6 https://github.com/tseemann/snippy, accessed on 28 February 2022) was used to identify core-genome single nucleotide polymorphisms (cgSNPs), together with the strain HS11286 (GenBank accession no. ASM24018v2) as a reference genome. WGSBAC finally used RAxML (Randomized Accelerated Maximum Likelihood) v8 [35] to reconstruct a phylogenetic tree based on the cgSNP alignment and the interactive Tree of Life (iTOL) v4 web tool (https://itol.embl.de/login.cgi, accessed on 28 February 2022) for visualization [36].

### 2.4. Data Availability

All study data are included in the article and supporting information. The data have also been submitted to the European Nucleotide Archive (ENA). The project accession number is PRJEB45776.

## 3. Results

### 3.1. Whole Genome Sequencing Data and MLST Analysis

Genome sequencing of twenty-four *K. pneumoniae* isolates yielded an average total number of 1,855,429 reads per isolate (range 1,597,112–2,155,888 Table S1). The isolates’ mean coverage was 86-fold (range from 73-fold to 95-fold). At the genus level, the first match (largest percentage proportion) for all isolates was always “*Klebsiella*” which was the classification for 91.52% of the reads on average (max 94.58%, min 86.57%). At the species level, the first match for all 24 isolates was always “*K. pneumoniae*.” Genome assembly yielded a genome size with a minimum of 5.305.557 bp and a maximum of 5.770.949 bp. The GC content was on average 57.08%. The mean N50 of the 24 assembled genomes was 27,275 bp (range 154,040–399,650 bp) (Table S1).

**Figure 1:**
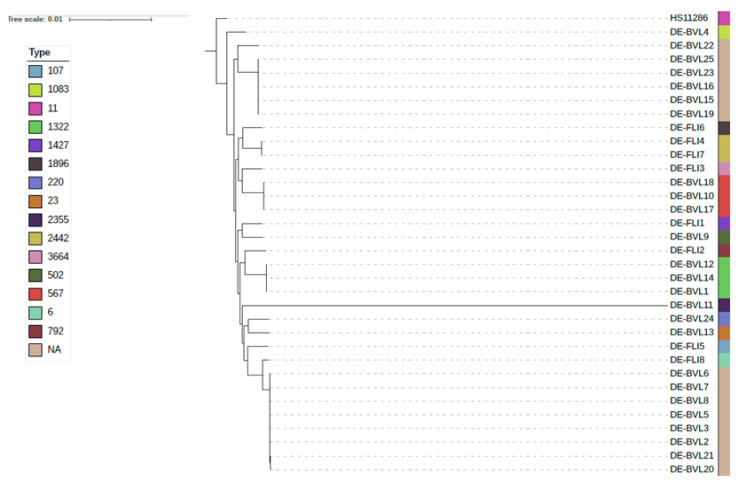
Phylogenetic tree using core-genome SNP of *K. pneumoniae* strains recovered from milk powder samples produced in Germany between 2005–2011.

MLST analyzes of the 24 *K. pneumoniae* isolates allocated ten strains into a distinct sequence type (ST) and 14 strains failed to be assigned due to new alleles. Three strains were assigned to ST/1322, three were assigned to ST/567 and one isolate each to ST/23, ST/220, ST/502 and ST/1083. Of the 14 strains (58%) which initially could not be assigned to a distinct sequence type, five strains isolated in 2007, 2008, 2010 and 2011 were allocated to novel STs (New 1), and later on assigned to ST/5625. One isolate from 2010 was another novel ST (New 2), which is assigned to ST/6016. The remaining eight isolates, which were isolated in 2005, 2006 and 2009, showed the occurrence of new alleles, and were near to ST/299 with the presence of new alleles with less than 100% identity emerged in the locus *pgi* (New 3), and later on were assigned to ST6014 (Table 1). The phylogenetic analysis using core-genome SNP calling included additional eight *K. pneumoniae* isolates from our collection (DE-FLI1 to DE-FLI8). The resulting phylogenetic tree implies that the six strains of unknown ST are closely related to ST/1083 (DE-MRI4), while the eight strains with untyped ST are similar to ST 6 (DE-FLI8), as shown in Figure 1.

### 3.2. Phenotyping and AMR Determinants in K. pneumoniae Isolates

Antibiotic susceptibility testing of the 24 *K. pneumoniae* strains revealed that all strains were susceptible to 14 of 18 tested antibiotics, i.e., ciprofloxacin, levofloxacin, amikacin, the tetracycline derivative tigecycline, trimethoprim/sulfamethoxazole, cefepime, cefotaxime, ceftazidime, ceftazidime/avibactam, ceftolozane/tazobactam, piperacillin/tazobactam, imipenem, meropenem and ertapenem in-vitro. Resistance to colistin and fosfomycin was seen for isolates DE-MRI3 and DE-MRI17 obtained in 2005 and 2007, respectively. One-third (8 out of 24) of the isolates displayed resistance to piperacillin, and four isolates showed non-susceptibility (one resistant and three intermediate) to chloramphenicol.

No genes mediating resistance to aminoglycosides were found. All strains harbored at least one variant of the sulfhydryl variable (*bla*_SHV_) ambler class A β-lactamase. Seven *bla*_SHV_ variants were identified, and *bla*_SHV-187_ was the most prevalent and found in 12 (50%) isolates, followed by *bla*_SHV-11_, *bla*_SHV-1_ and *bla*_SHV-145_, which were found in ten, nine and eight isolates, respectively. The variants of *oqx*A and *oqx*B, conferring resistance to phenicol/quinolone, were found in all isolates, despite only four isolates showing non-susceptibility to chloramphenicol. The *oqx*B17 variant was the most prevalent found in elven isolates, followed by *oqx*A7 and *oqx*A10, found in six isolates, while *oqx*B25 and *oqx*B26 were found in the same five isolates. The two genes *pmr*B_R256G and *mcr-*9 conferring resistance to colistin were found in two different isolates obtained from milk powder taken in 2005 and 2009, respectively. However, both isolates were susceptible to colistin. In contrast, the colistin-resistant strain did not carry any genes from the *mcr* family. More than half of the isolates, 67% (*n* = 16), harbored the *fos*A gene, which confers reduced susceptibility to fosfomycin; however, only one isolate was resistant to fosfomycin. Moreover, all isolates harbored genes for the antibiotic efflux pump, i.e., *ram*A the positive regulator of AcrAB-TolC, the *emr*R and *emr*D genes, *Klebsiella*_*pneumoniae*_KpnE, KpnF, KpnG, KpnH; *Klebsiella*_*pneumoniae*_*acr*A, *acr*B, *acr*D and *Klebsiella*_*pneumoniae*_OmpK37 (Table S1).

### 3.3. Characterization of Plasmid Replicons and Virulence-Associated Genes in K. pneumoniae

The plasmid replicons in the *K. pneumoniae* isolates were analyzed using Plasmid Finder. Ten different types of plasmid replicons were identified in the 24 *K. pneumoniae* isolates. Four replicons belonged to the IncF plasmid family, three belonged to Col-type plasmids, two belonged to the Inc-type plasmid family and one belonged to the *rep*A. All isolates were found to be carrying plasmid replicons; however, the number of replicons was variable in each strain, and ranged from one to six. The strain ST502 (DE-MRI9) carried the highest number of plasmid replicons (*n* = 6) among the strains and contained the second-highest number of virulence-associated genes (*n* = 21) after the ST23 strain, which carried 29 virulence factors. The IncFIB(K)-1-Kpn3 plasmid replicon was predominant in 16 (67%) isolates, followed by IncFII_1_pKP91 and Col440I_1 that were found in 13 (54%) and 11 (46%) isolates, respectively. The IncFIB.Mar.-1-pNDM.Mar and IncHI1B-1-pNDM.MAR replicons were found in seven isolates, while the IncFIB.pKPHS1.-1-pKPHS1 replicon was found in four isolates and IncR-1 was found in three isolates. The ColRNAI-1 was found in two isolates, while Col440II_1 and RepA_1_pKPC.CAV1321 were found once (Table S1).

In the current study, 29 virulence-associated genes were identified in the 24 strains. The majority of identified virulence-associated genes belonged to the Iron uptake siderophores. The prototypic catecholate siderophore enterobactins (*ent*B, *fep*C) were found in all isolates (100%), while *ent*A was found in 21 (87.5%) isolates. Eleven phenolate siderophore yersiniabactin-related genes (*ybt*A, *ybt*E, *ybt*P, *ybt*Q, *ybt*S, *ybt*T, *ybt*U, *ybt*X, *irp*1, *irp*2, *fyu*A) were detected and all were found in the same two (8%) isolates. The citrate-hydroxamate siderophore aerobactins (*iuc*A, *iuc*B, *iuc*C, *iut*A), the glycosylated salmochelins (*iro*B, *iro*C, *iro*D, *iro*N) and the regulator of mucoid phenotype A (*rmp*A2, *rmp*A, *rmp*C and *rmp*D) were found only in an ST23 strain (4%). The outer membrane protein A (*omp*A), which is related to the T6SS-II secretion system of *Klebsiella* and further six adherence virulence genes related to ECP from *E. coli* (*ykg*K.ecpR, *yag*V.ecpE, *yag*W.ecpD, *yag*X.ecpC, *yag*Y.ecpB, *yag*Z.ecpA) were found in all isolates (100%) (Table S1). One ST23 strain (DE-MRI13) isolated in 2007 was susceptible to all tested antibiotics and harbored determinants discriminatory for hypervirulent *K. pneumoniae* (hvKp), e.g., aerobactin, salmochelin, yersiniabactin, enterobactin genes and regulator of mucoid phenotype A, while it was hypermucoviscosity negative and devoid of *mag*A and *wca*G genes (Table 2). Plasmids of the ST/23 strain (DE-MRI13) were compared with the pK2044 plasmid of an ST/23 isolate NC_006625 available at https://www.ncbi.nlm.nih.gov/nuccore/NC_006625.1 (accessed on 28 February 2022) regarding the content of *rmp*A/A2. Our ST/23 isolate (DE-MRI13) contains two contigs harboring INK factors. Contig 24 is 63.276bp long and harbours IncFIB(K)_1_Kpn3 and contig 20 is 84.546 bp long and contains IncHI1B_1_pNDM-MA. The *rmp*A2 was found only on the contig 20 of DE-MRI13 strain, while *rmp*A was found on the plasmid pK2044 of strain NC_006625 from NCBI.

The plasmid carrying the *mcr*-9 gene in the present study was compared with the plasmids pA2483 and pA2504 harboring *mcr-*9 found in carbapenem-resistant and colistin non-resistant *Enterobacter cloacae* complex isolates recently published by Kananizadeh et al. in 2020 [37]. The plasmids pA2483 and pA2504 are 288.696 bp and 276.927 bp in size, respectively. Both contain three INK factors: IncHI2A, IncHI2 and RepA. In the present study, the strain (DE-MRI21) harbours *mcr*-9 on contig 37. This contig exclusively contains the INK factor RepA and is 31.103 bp in size. Regarding genetic factors for AMR, the plasmid carrying the contig 37 contains exclusively *mcr*-9, while the other plasmids contain additional AMR genes conferring resistance to heavy metals.

## 4. Discussion

*Klebsiella pneumoniae* is a notorious pathogen throughout the health care system worldwide. It causes a wide range of severe infections that are often difficult to treat [38]. In animals, it has been implicated in bovine mastitis [6], as well as in mortalities among affected cows [7,8], resulting in substantial economic losses in the dairy industry [39]. *K. pneumoniae* was among the most frequently isolated bacterial species in milk substitution formulas for infants collected from 35 countries [40]. A high incidence of MDR strains has been reported in pasteurized milk and whole milk powder samples collected from retail shops in Mexico, representing a public health hazard [41]. ESBL-producing *K. pneumoniae* was isolated from food handlers who consumed unpasteurized milk and raw meat [42]. To assess whether the powdered milk might be an essential source for disseminating *K. pneumoniae* or resistance genes in Germany, we investigated 24 isolates obtained from the milk powder of powdered milk producers by WGS. Eight of the strains displayed resistance to piperacillin, one to colistin and one to fosfomycin. All isolates were susceptible to ciprofloxacin and levofloxacin (fluoroquinolones), amikacin (aminoglycoside), the tetracycline derivative tigecycline, trimethoprim/sulfamethoxazole, 3rd generation cephalosporins cefotaxime, ceftazidime, ceftazidime/avibactam and ceftolozane/tazobactam, cefepime (4th generation cephalosporin), piperacillin/tazobactam and to the carbapenems (imipenem, meropenem and ertapenem). Colistin is considered a last-resort antibiotic and is used to treat infections caused by MDR Gram-negative bacteria [43]. Therefore, the isolation of a strain resistant to colistin from milk powder is alarming and carries a potential risk for humans, particularly for neonates and infants consuming dried milk. Although none of the isolates recovered from milk powder showed an MDR pattern, several AMR genes mediating resistance to ß-lactams, efflux pumps, phenicol/quinolone, colistin and fosfomycin were identified.

In the current study, all isolates were historical isolates recovered from milk powder samples between 2005–2011 and had not been investigated regarding resistance profile, MLST, AMR and virulence-associated genes at the time. The investigation of historical isolates by WGS provides a large amount of data, which can help to elucidate the development of AMR in Germany and previously unknown resistance mechanisms. All isolates were obtained from the end product of the manufacturing process. However, it was not possible to determine the source of contamination, i.e., human, animal or environmental. As the milk is pasteurized and heat treatment is applied during concentration and drying, the survival of *K. pneumoniae* and the introduction of strains from animal origin, i.e., cow, is highly unlikely. However, recontamination in the production plant via personnel, circulating air and packing material may cause contamination in the end product. Only a few articles exploring ST in human isolates have been published to date, and none on animal isolates. In the current study, an ST/23 strain, which is associated with a hypervirulent *K. pneumoniae* type (hvKp) was isolated in 2007. The isolate harbored determinants discriminatory for hypervirulent strains, e.g., aerobactin, salmochelin, yersiniabactin, enterobactin and regulator of mucoid phenotype A genes; however, it was hypermucoviscosity negative and devoid of *mag*A and *wca*G genes. An ST/23 strain producing OXA-48 was isolated previously from tracheal secretions of hospitalized patients [44]. A hvKp ST268 strain was also isolated from water in northern Germany [45] and an hvKp ST2398 strain was isolated from a patient with liver abscess and endophthalmitis in 2016 [46]. An MDR ST/11 strain was reported in European mouflons (*ovis orientalis musimon*) in 2016 [47].

Carbapenems and colistin are considered the drugs of choice for treating MDR bacterial infections. Infections caused by carbapenemase-producing *K. pneumoniae* are increasing in Germany [48,49], and colistin-resistant strains carrying the *mcr*-1 gene were reported in clinical samples in 2017 [50], and from wastewater treatment plants [51]. In the current study, *bla*_SHV_ variants ß-lactamase were found in all strains. Since the late 20th century, *bla*_SHV-11_ and *bla*_SHV-5_ are among the most frequently detected genes in *K. pneumoniae* of human origin in Germany [52]. The *bla*_SHV_ variants were also identified in *K. pneumoniae* strains isolated from European mouflons [47], pet animals [53] and environmental samples collected from pig farms [54]. Although *bla*_OXA-48_ and *bla*_CTX-M-15_ are the most frequently identified resistance genes in *K. pneumoniae* strains isolated from patients [15,18,55] and pet animals [56,57] in the last decade in Germany, none were found in the current study. The presence of pmrB_R256G and *mcr*-9 genes did not confer colistin resistance in our samples. The *mcr*-9 is a mobilized and plasmid-mediated colistin resistance gene identified in *Salmonella enterica* [58]. It is the most widely disseminated gene of the *mcr*-family after *mcr*-1, identified in isolates from 40 countries across six continents [59]. Its ability to cause colistin resistance has been investigated in several studies, and the occurrence of two different frameshift mutations in *mcr*-9 probably leads to non-functional *mcr*-9 proteins [58]. However, the occurrence of frameshift mutation was not investigated in the *mcr*-9 positive strain. To the best of our knowledge, the *mcr*-9 was not reported in *K. pneumoniae* strains isolated from humans, veterinaries and the environment in Germany. However, colistin-resistant strains carrying the *mcr*-1 gene have been reported in clinical samples from leukemia patients [50] and wastewater treatment plants [51]. Our results agree with Kananizadeh and coworkers, who found colistin-susceptible *Enterobacter cloacae* harboring *mcr*-9 [37]. Comparison of the plasmids carrying *mcr*-9 in our study and the plasmids from Kananizadeh et al. [37] revealed that our strain harbored exclusively *mcr*-9 on contig 37. This contig exclusively contains INK factor RepA, while the plasmids described by Kananizadeh contain three INK factors, IncHI2A, IncHI2 and RepA, and contain additional AMR genes such as *pco*S conferring resistance to Arsenic and *ter*D, *ter*Z and *ter*W conferring resistance to Tellurium.

Of the 24 isolates, only one was resistant to fosfomycin, despite the presence of *fos*A in 16 isolates. Chromosomal *fos*A genes confer a high level of resistance to fosfomycin in Gram-negative bacteria [60]. However, it failed to mediate resistance in the current *K. pneumoniae* isolates. The *fos*A gene is widely distributed on the chromosomes of many Gram-negative bacteria. Fosfomycin has become a valid choice against MDR, extended-spectrum ß-lactamases (ESBL) and carbapenem-resistant *Enterobacteriaceae*, and it is recommended as an emerging treatment for infection caused by MDR bacterial pathogens [61,62]. Recently, it was reported that FosA inhibitors such as sodium phosphonoformate (PPF) could restore the activity of fosfomycin in chromosomally encoded FosA Gram-negative bacterial strains, including *K. pneumoniae* [63]. Moreover, a small-molecule inhibitor of FosA significantly potentiated fosfomycin activity against Gram-negative pathogens harboring the *fos*A gene, including *K. pneumoniae* [64]. Some of these FosA inhibitors are clinically approved antiviral agents. Exposure of *K. pneumoniae* strains to such inhibitors could explain the susceptibility of the strains to fosfomycin despite harboring the *fos*A gene.

Four main components are known to mediate infectivity and pathogenesis in *K. pneumoniae,* including adherence factors, siderophore activity, O-lipopolysaccharide (LPS) and K-capsular antigens [20]. Six adherence-related genes belonging to ECP (*E. coli* common pilus) were found in all strains; however, no Type I fimbriae was found. ECP is a pilus of EHEC O157:H7, necessary for the initial attachment to host epithelial cells and subsequent colonization [65]. Iron acquisition systems or siderophores are needed in *K. pneumoniae* to overcome host defense and for deep penetration of the tissues [66]. The majority of identified virulence genes belonged to iron uptake siderophores. Enterobactins were found in almost all isolates, while salmochelin, aerobactin and the regulator of mucoid phenotype A were found in one isolate and yersiniabactin in two. The siderophore aerobactin is a critical and dominant virulence factor produced by hypervirulent *K. pneumoniae* [67]. hvKp infections are currently increasing, and are considered a global threat [68,69]. Several virulence genes were found to contribute to the hypervirulent phenotype, including mucoviscosity-associated gene A (*magA*) and (*wcaG*) and regulator of mucoid phenotype A (*rmpA* and *rmpA2*) [70,71]. The *rmp*A2/A are highly associated with hvKp as they positively regulate the *cps* locus during the capsular polysaccharide synthesis, resulting in the hypercapsule phenotype [72], and were identified in the ST/23 strain in the current study. However, recent studies showed that aerobactin is the major hvKp-specific virulence factor [73,74] and is used to differentiate hvkp either alone or combined with salmochelin and *rmp*A2 [75], or with hypermucoviscosity [76]. The role of yersiniabactin in pathogenesis is still unknown [77]. However, it was found in 17.7% of *K. pneumoniae* strains isolated from blood cultures and urine from hospitalized patients in Munich [78].

Plasmids are the major factors included in the dissemination of pathogenicity and resistance-associated genes in bacteria. Their duplication and survival capacity depends on their replication determinants (replicon) [79]. The plasmid and replicons content in *K. pneumoniae* isolates were investigated. Plasmids were identified in almost all *K. pneumoniae* strains with a diverse replicon content. The IncF plasmid family members are the most frequent plasmid types in *Enterobacteriaceae* and were found in all strains. More than half of the isolates contained IncFIB and IncFII plasmids. IncF plasmids play a significant role in disseminating AMR, and are often associated with virulence genes in *Enterobacteriaceae* [80]. Although the IncF plasmids have been associated with the emergence of *bla*_CTX-M-15_ globally [81,82], none of the isolates in the current study harbored *bla*_CTX_ variants. Two Inc-type plasmids were found, IncHI1B and IncR. The IncR plasmid is often responsible for the horizontal transmission of *bla*_KPC_ to other *Enterobacteriaceae* by conjugation [1]. Half of the isolates contained the Col440I-type plasmid replicon, and two isolates contained ColRNAI. Resistance to carbapenems in XDR *K. pneumoniae* due to the *bla*_NDM-1_ is mediated by the acquisition of multi-replicons containing ColRNAI, IncFIB Col440I, IncFII and IncFIB plasmid [83], and all have been identified in the current study.

## 5. Conclusions

*K. pneumoniae* isolates recovered from the end of the production process of powdered milk samples in Germany revealed a wide variety of AMR genes, mostly mediating resistance to ß-lactams and virulence-associated genes, which were mostly siderophores. The isolates showed susceptibility to the majority (14 of 18) of tested antibiotics. The appearance of isolates resistant to colistin and fosfomycin, chloramphenicol and piperacillin is alarming. Plasmids are known to host virulence and antibiotic resistance coding genes. The presence of a wide variety of such plasmids in dried milk samples highlights the threat that can occur from consuming contaminated milk powder, and is considered a threat to public health. To the best of our knowledge, *mcr*-9 has not been isolated from *K. pneumoniae* in Germany, and further investigations of the occurrence of frameshift mutation are required in the *mcr*-9 positive strain. Additional studies on a large number of recently isolated strains applying conjugation or transformation assays are required to evaluate the AMR gene transferability.

## Figures and Tables

**Table 1:** Description of the typed and novel STs and allelic profiles of the untyped *K. pneumoniae* strains recovered from milk powder samples produced in Germany between 2005–2011.

STs	ID	Origin	Year	*gap*A	*inf*B	*md*h	*pgi*	*pho*	*rpo*B	*ton*B
1322	DE-MRI1	Milk powder	2005	2	5	121	8	180	1	6
	DE-MRI12	Milk powder	2007	2	5	121	8	180	1	6
	DE-MRI14	Milk powder	2007	2	5	121	8	180	1	6
567	DE-MRI10	Milk powder	2006	2	1	77	1	17	4	42
	DE-MRI17	Milk powder	2007	2	1	77	1	17	4	42
	DE-MRI18	Milk powder	2008	2	1	77	1	17	4	42
23	DE-MRI13	Milk powder	2007	2	1	1	1	9	4	12
220	DE-MRI24	Milk powder	2011	2	1	2	1	45	4	9
502	DE-MRI9	Milk powder	2003	2	53	3	1	10	4	18
1083	DE-MRI4	Milk powder	2005	2	1	2	1	13	1	23
New 1 5625	DE-MRI15	Milk powder	2007	2	1	1	3	40	1	22
	DE-MRI16	Milk powder	2007	2	1	1	3	40	1	22
	DE-MRI19	Milk powder	2008	2	1	1	3	40	1	22
	DE-MRI23	Milk powder	2010	2	1	1	3	40	1	22
	DE-MRI25	Milk powder	2011	2	1	1	3	40	1	22
New2 6016	DE-MRI22	Milk powder	2010	2	6	3	1	1	102	25
New3 6014	DE- MRI 2	Milk powder	2005	2	10	1	393	56	24	31
	DE- MRI 3	Milk powder	2005	2	10	1	393	56	24	31
	DE-MRI 5	Milk powder	2005	2	10	1	393	56	24	31
	DE-MRI 6	Milk powder	2006	2	10	1	393	56	24	31
	DE-MRI 7	Milk powder	2006	2	10	1	393	56	24	31
	DE-MRI 8	Milk powder	2006	2	10	1	393	56	24	31
	DE-MRI20	Milk powder	2009	2	10	1	393	56	24	31
	DE-MRI21	Milk powder	2009	2	10	1	393	56	24	31

ST: Sequence type.

**Table 2:** Genetic features of 24 *K. pneumoniae* isolates originating from milk powder samples.

Parameters of Twenty-Four *K. pneumoniae* Isolates	
Average total number of reads	18,554,295 reads per isolate
Isolates mean coverage	8558-fold
**Genome**	
Minimum genome size (bp)	5.305.557 bp
Maximum genome size (bp)	5.770.949 bp
The average of the GC content (%)	57.08%
The mean N50	27,275 bp
**Database accession no.**	
European Nucleotide Archive (ENA)	Project accession number: PRJEB45776
**Virulence associated genes (% coverage/No. of isolates)**	
Enterobactin (Iron uptake)	*ent*A (99.20/21); *ent*B (99.18/24); *fep*C (94.61/24)
Salmochelin (Iron uptake)	*Iro*B (97.08/1); *iro*C (99.28/1); *iro*D (100/1); *iro*N (98.81/1). All were found in the same isolate.
Aerobactin (Iron uptake)	*iuc*A (99.72/1); *iuc*B (100/1); *iuc*C (99.89/1); *iut*A (99:96/1). All were found in the same isolate.
Yersiniabactin (Iron uptake)	*ybt*A, *ybt*E, *ybt*P, *ybt*Q, *ybt*S, *ybt*T, *ybt*U, *ybt*X, *irp*1, *irp*2, *fyu*A. All were found with 100% coverage in the same two isolates.
T6SS-II	*omp*A (100/24).
Adherence (Gene related to ECP from *E. coli*)	ykgK.ecpR (98.31/24); yagV.ecpE (99.74/24); yagW.ecpD (100/24); yagX.ecpC (99.96/24); yagY.ecpB (100/24); yagZ.ecpA (99.32/24).
Regulator of mucoid phenotype A	*rmp*A2 (96.67/1); *rmp*A (98.57/1); *rmp*C (90.23/1); and *rmp*D (100/1).
**Antibiotic resistance determinants**	
Resistance genes (%)	*bla*_SHV_ (100); *oqx*A (100); *oqx*B (100); *Kpn*E (100); *Kpn*F (100); *Kpn*G (100); *Kpn*H (100); Ompk37 (100); *acr*A (100); *acr*B (100); *acr*D (100); *emr*R (100); *emr*D (100); *ram*A (100); *fos*A (71); *pmr*B_R256G (4); *mcr-*9 (4).
Plasmid replicons	Col family (Col440II; Col440I; ColRNAI); IncF family (IncFIB.K.; IncFIB.Mar.; IncFIB.pKPHS1.; IncFII_1_pKP91); Inc family (IncHI1B and IncR); Rep A family (RepA_1).

## Data Availability

All study data are included in the article and supporting information. The data have also been submitted to the European Nucleotide Archive (ENA). The project accession number is PRJEB45776.

## Supplementary Materials

The following supporting information can be downloaded at: https://www.mdpi.com/article/10.3390/microorganisms10030564/s1, Table S1: The average read length and the coverage, data of genome assembly, the results of antibiotic susceptibility testing, metadata, MLST, AMR and virulence-associated genes and plasmid replicons of 24 *K. pneumoniae* isolates from milk powder samples produced in Germany.
